# Using Robot-Based Variables during Upper Limb Robot-Assisted Training in Subacute Stroke Patients to Quantify Treatment Dose

**DOI:** 10.3390/s22082989

**Published:** 2022-04-13

**Authors:** Pascal Jamin, Christophe Duret, Emilie Hutin, Nicolas Bayle, Typhaine Koeppel, Jean-Michel Gracies, Ophélie Pila

**Affiliations:** 1 Institut Robert Merle d’Aubigné, Rééducation et Appareillage, 94460 Valenton, France; p.jamin@irma-valenton.fr; 2 Centre de Rééducation Fonctionnelle Les Trois Soleils, Médecine Physique et de Réadaptation, Unité de Neurorééducation, 77310 Boissise-Le-Roi, France; c.duret@les-trois-soleils.fr (C.D.); typhaine.koeppel@gmail.com (T.K.); 3 Laboratoire Analyse et Restauration Du Mouvement (ARM), Hôpital Henri MONDOR, Université Paris-Est, 94000 Créteil, France; emilie.hutin@aphp.fr (E.H.); nicolas.bayle@aphp.fr (N.B.); jean-michel.gracies@aphp.fr (J.-M.G.); 4 Bioingénierie, Tissus et Neuroplasticité (BIOTN), Université Paris-Est Créteil, 94000 Créteil, France

**Keywords:** hemiparesis, robotics, upper extremity, intensity, neurorehabilitation

## Abstract

In post-stroke motor rehabilitation, treatment dose description is estimated approximately. The aim of this retrospective study was to quantify the treatment dose using robot-measured variables during robot-assisted training in patients with subacute stroke. Thirty-six patients performed fifteen 60 min sessions (Session 1–Session 15) of planar, target-directed movements in addition to occupational therapy over 4 (SD 2) weeks. Fugl–Meyer Assessment (FMA) was carried out pre- and post-treatment. The actual time practiced (percentage of a 60 min session), the number of repeated movements, and the total distance traveled were analyzed across sessions for each training modality: assist as needed, unassisted, and against resistance. The FMA score improved post-treatment by 11 (10) points (Session 1 vs. Session 15, *p* < 0.001). In Session 6, all modalities pooled, the number of repeated movements increased by 129 (252) (vs. Session 1, *p* = 0.043), the total distance traveled increased by 1743 (3345) cm (vs. Session 1, *p* = 0.045), and the actual time practiced remained unchanged. In Session 15, the actual time practiced showed changes only in the assist-as-needed modality: −13 (23) % (vs. Session 1, *p* = 0.013). This description of changes in quantitative-practice-related variables when using different robotic training modalities provides comprehensive information related to the treatment dose in rehabilitation. The treatment dose intensity may be enhanced by increasing both the number of movements and the motor difficulty of performing each movement.

## 1. Introduction

Upper limb paresis is the most frequent sequel of a stroke and has a major functional impact on autonomy and quality of life [1]. Up to 70% of the patients do not recover upper limb function 6 months after a stroke [2,3,4,5]. Improvement of upper limb function is thus a crucial issue for both clinicians and patients.

Knowledge of neuroplasticity [6,7] and the fact that motor learning may be preserved after a stroke [6,8,9,10,11,12] led to a real change in the delivery of neurological rehabilitation. Movement repetition [13,14,15] and intensity [16,17,18], along with the level of effectiveness of the rehabilitation technique or program [19], are essential to driving motor learning.

The definition of a treatment dose in the field of rehabilitation has been widely debated in recent years but has not yet been resolved [20,21,22,23]. In pharmacology, the active components and metabolism of medications are known. Therefore, dose modulation is simple [23]. Repetition and intensity have been clearly identified as relating to the rehabilitation dose, but their description and quantification remain limited [24]. In the field of rehabilitation, a new framework was recently proposed by Hayward et al. to establish a standardized approach to the definition of a treatment dose by providing a description of the different dimensions of a dose for non-pharmacological interventions [25]. This enriched framework was designed to collect information from both the peripheral dimensions of a dose (treatment period) and the internal dimensions (task duration, task difficulty, and task intensity). However, collecting such data, particularly for the internal dimension of a dose, is time consuming and thus could increase costs.

Technological systems designed for rehabilitation, such as robotic devices and virtual reality systems, fulfil the criteria required for motor learning [26]. However, the evidence supporting their use for improving upper limb function is weak. Large meta-analyses of trials performed since the 1990s have shown that robotic devices can reduce motor impairment in both the subacute and chronic phase of a stroke. However, the impact on upper limb function is less obvious [27,28,29]. The recent RATULS trial included 770 patients and compared rehabilitation with a robotic device, conventional rehabilitation (occupational therapy and physiotherapy), and enhanced upper limb therapy [30]. Enhanced upper limb therapy resulted in significant improvements in upper limb function, while robot training did not. However, in the robot group, impairment reduced in the muscle groups that were trained by the robot and this improvement was maintained over time [30]. The conclusions of this trial are, therefore, not entirely negative; however, the challenge remains to translate the reduction in impairment into improvements in function [31].

One advantage of robotic devices is that they can easily, objectively, and automatically record variables that are difficult to measure in clinical practice, such as the duration of active participation and the number of repetitions [32]. The disadvantage is that the level of motor difficulty achieved during the sessions is missing. Motor difficulty can be modulated using the different training modalities available in the robotic system. These data can provide an objective indication of the motor difficulty of a task; in conventional therapy, the measurement of task difficulty is usually limited to the subjective impression of the patient and the therapist.

The aim of this retrospective study was to establish a comprehensive description of the internal dimension of the dose related to upper limb robot-assisted therapy (RT) in a sample of patients with hemiparesis in the subacute phase of a stroke. To accurately characterize the administered treatment dose with the robotic device, changes across robotic sessions were analyzed using three quantitative parameters: the actual time patients spent in practice (actual practice time), the number of repeated movements performed, and the total distance traveled by the hand. Difficulty in performing motor tasks with the robotic device was quantified, using these parameters, depending on the physical training modalities offered by the robot.

## 2. Materials and Methods

### 2.1. Participants

This retrospective study included data from inpatients in the subacute phase of a stroke who were undergoing rehabilitation in the rehabilitation department of the Centre de Réadaptation Fonctionnelle (CRF) Les Trois Soleils (Boissise-le-Roi, France) between 2009 and 2019. The study was performed in accordance with current French legislation (reference N°004 (MR004)) and was granted approval by our internal ethics committee in line with the Data Protection Act [33]. It was registered on the Health Data Hub (N° F20211012141808).

Patients were included if: (1) they were aged over 18 years and not under legal protection; (2) they had hemiparesis following a single, unilateral, ischemic, or hemorrhagic stroke; (3) they had completed 19 ± 4 sessions of upper limb robotic rehabilitation with the InMotion Arm^®^ (Watertown, MA, USA) between 4 and 23 weeks post-stroke (i.e., subacute phase); (4) they had undergone Fugl–Meyer Assessment and robot-based evaluation (point-to-point) performed ±6 days before and after the intervention; (6) they had spent less than 30% of RT session time on games (non-therapeutic exercises); and (7) their therapist had complete rehabilitation notes documented for RT.

Patients were not included if: (1) they had been administered anti-spasticity medication during or before the RT period and (2) they had used another technological rehabilitation device before or during the RT period.

**Figure 1:**
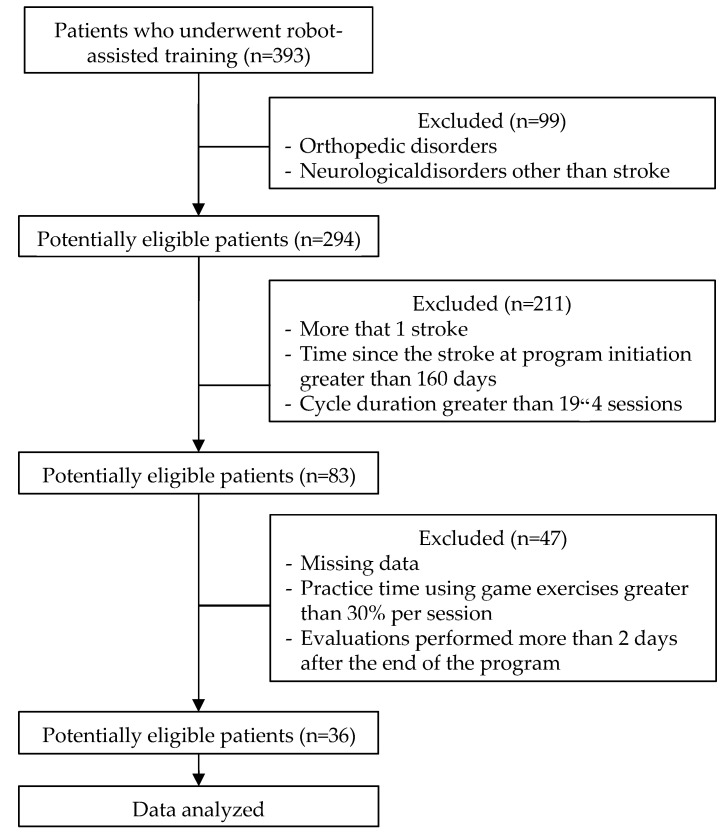
Flow diagram of the criteria for patient inclusion.

Data from 393 eligible patients who had participated in RT between 2009 and 2019 were screened; 36 patients meeting the inclusion criteria were included (Figure 1); the mean (SD) age was 59 (16) years, the mean time since the stroke at program initiation was 54 (26) days, the mean duration of the RT intervention was 31 (11) days, and the mean initial FMA score was 23 (17) points (Table 1).

### 2.2. Robotic System

The InMotion Arm^®^ robot (InMotion 2, Interactive Motion Technologies, Inc., Watertown, MA, USA) is a 2 degrees of freedom planar distal-effector-type manipulator that trains shoulder and elbow movement in the horizontal plane; an impedance controller is incorporated in the device, and it has low intrinsic inertia. Sensors continuously record the kinematic and kinetic parameters of the hand movements performed by the patient during the session (position, force, and time). These data are used to provide visual feedback of performance and can also be used to analyze variables of interest. The robotic device is equipped with a comfortable chair that is mounted on rails to allow adjustment. The distance between the screen and the patient and the height of the seat are also adjustable. A thoracic harness prevents compensatory trunk movements. The patient’s forearm rests in a trough in which the wrist is held in a neutral position. The fingers are flexed around a vertical handle (end-effector) and are held in place by straps if necessary. Ten minutes of the 60 min session is spent setting up, providing instructions, and removing the patient from the device.

### 2.3. Interventions

The upper limb rehabilitation program involved 60 min/day of RT using the InMotion Arm^®^ device and 60 min/day of conventional occupational therapy, five times per week, for 4 weeks. Conventional occupational therapy sessions included mobilization and stretching of the paretic upper limb to improve or maintain a joint range of motion, individual joint and whole upper limb exercises, fine motor control and grasping, exercises to improve sensation, and functional and fun exercises involving both motor and cognitive functions. RT sessions were divided into three phases:

- A warm-up phase involving games (phase 1);
- A training phase based on exercises (phase 2);
- A relaxation phase involving games (phase 3).

**Figure 2:**
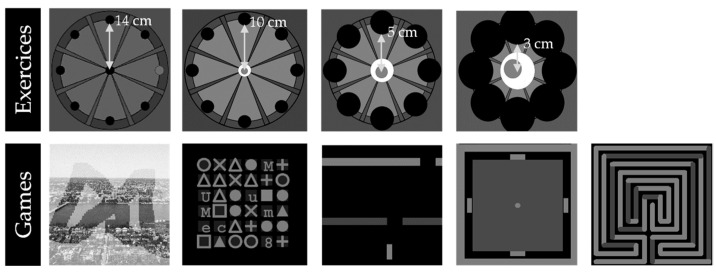
User interfaces for exercises used in Phase 2 and games used in Phase 1.

The user interfaces for games and exercises are displayed in Figure 2. The exercises (Phase 2) are a greater practice time than the games. The display for the exercises is composed of 8 targets distributed around a 14 cm radius circle, and the starting position is in the center. The therapist sets the number of movement repetitions and distance to be covered to reach the target (3/5/10/14 cm in all directions; Figure 2). The training modality affects the patient’s motor skills by increasing the difficulty level for performing the exercise. There are three:

- Assist-as-needed modality: The patient has to reach the target with assistance tailored into the performance;
- Unassisted modality: The patient has to reach the target without any assistance;
- Against resistance: The patient has to reach the target against a resistance.

The therapist thus chooses the training modality that ensures that the exercises are always challenging. The progression of the difficulty is, therefore, from the assist-as-needed modality to unassisted and then against resistance. The level of therapist control over the games is lower, and fewer data are recorded. Therefore, the intensity of the games cannot be accurately determined.

### 2.4. Clinical Evaluation Procedure Pre- and Post-Intervention

Upper limb impairment was evaluated before and after the intervention. Impairment was evaluated using the upper limb section of the Fugl–Meyer Assessment (FMA). This tool is reliable and sensitive to change and has been validated for use in spastic paresis in the subacute phase of a stroke [34,35,36]. The upper limb section is scored out of 66 points, and higher scores indicate lower levels of impairment.

### 2.5. Data Collected during Interventions

Only data from RT sessions were collected as data from occupational therapy were not available. For each game (Phases 1 and 3), only the actual practice time (normalized to a session length of 60 min) was collected, the level of therapist control over the games was lower, and fewer data were recorded. The actual practice time in games and in exercises represented the time during which patients were active; the remaining time represented inactive time. During Phase 2, for each RT session, several exercises were performed with various training modalities. Thus, for each exercise, the following data were collected:

- The number of movements performed by the patient in each training modality used (assist as needed, unassisted, or against resistance);
- The actual practice time in each training modality used (assist as needed, unassisted, or against resistance (in min);
- The distance to be covered to reach the target used (defined by the therapist before each exercise).

Then, the total distance traveled parameter (in cm) was calculated for each RT session as the sum of the product of the distance to be covered to reach the target used and the number of movements performed for each exercise.

Based on these data, three robot-based variables were analyzed per RT session:

- The mean number of repeated movements in each training modality used;
- The mean actual practice time in each training modality used (in percentage; normalized to a session length of 60 min);
- The mean total distance traveled in each training modality used (in cm).

### 2.6. Statistical Analysis

Student’s *t*-test was used to analyze the effects of the intervention on the FMA score. Repeated measures analysis of variance (ANOVA) was used to analyze change across the sessions for three robot-based variables (mean number of repeated movements, mean actual practice time, and mean total distance traveled). Session (Session 1–Session 15) * modality (assist as needed, unassisted, or against resistance) interactions were analyzed based on these variables using the two-factor ANOVA. A Bonferroni correction was applied for multiple comparisons. Significance was set to *p* < 0.05, and SPSS 17.0 was used for all analyses.

## 3. Results

### 3.1. Clinical Outcomes Pre- and Post-Intervention

Changes in the FMA score and sub-scores are summarized in Table 2. From pre- to post-intervention, the FMA score improved by a mean 11.2 (SD 9.6) points (+17%, *p* < 0.001). Shoulder/elbow, wrist, hand, and coordination velocity sub-scores also increased: +6.3 points (+18%, *p* < 0.001); +1.7 points (+17%, *p* < 0.001); +2.6 points (+19%, *p* < 0.001), and +0.5 points (+9%, *p* = 0.013), respectively.

### 3.2. Practice Time during Robot-Assisted Therapy Session

**Figure 3:**
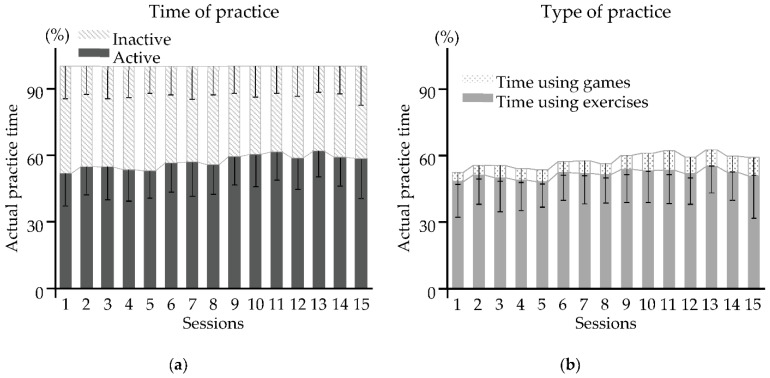
Active and inactive practice time over 15 sessions of RT: (**a**) Time of practice; (**b**) type of practice. Results are expressed as the mean (SD). Practice time (in %) was normalized to a session length of 60 min.

Results are summarized in Figure 3. With all sessions pooled, patients produced active movements for a mean 57 (14) % of each 60 min session, 6 (7) % of which involved games and 51 (14) % exercises.

### 3.3. Outcomes with All Robotic Training Modalities Pooled

Results are summarized in Table 3. From Session 1 to Session 15, changes were observed in the mean number of repeated movements (main effect, *p* < 0.001; Session 1 vs. Session 15, +46%, *p* = 0.008) and the mean total distance traveled (main effect, *p* < 0.001; Session 1 vs. Session 15, +140%, *p* < 0.001) but not in the mean actual practice time (main effect, ns). Changes occurred from the 6th session for the mean number of repeated movements (+38%, *p* = 0.043) and the mean total distance traveled (+100%, *p* = 0.045; multiple comparisons, Table 3).

### 3.4. Outcomes for Each Robotic Training Modality

**Figure 4:**
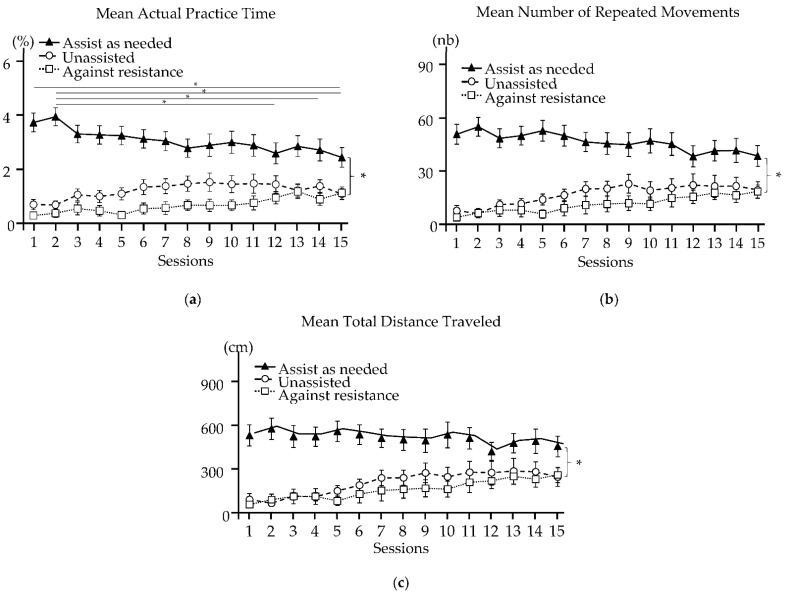
Robot-based variables measured during each training modality over 15 sessions of RT. Schemes follow another format. If there are multiple panels, they should be listed as: (**a**) Mean Actual Practice Time; (**b**) Mean Number of Repeated Movements; and (**c**) Mean Total Distance Traveled. Results are expressed as the mean (SEM). Parentheses and *, significance of the difference in the evolution between the three training modalities from Session 1 to Session 15; *, significance of the difference in the evolution between sessions (multiple comparisons).

The results are summarized in Figure 4. From Session 1 to Session 15, changes in the mean actual practice time (main effect, *p* < 0.001), the mean number of repeated movements (main effect, *p* ≤ 0.001), and the mean total distance traveled (main effect, *p* = 0.004) depended on the training modalities. Only the mean actual practice time changed significantly in the assist-as-needed modality: it decreased by 26% in Session 15 (vs. Session 1, *p* = 0.013), by 26% in Session 15 (vs. Session 2, *p* < 0.001), by 22% in Session 14 (vs. Session 2, *p* = 0.035), and by 26% in Session 12 (vs. Session 2, *p* = 0.005).

## 4. Discussion

The aim of this retrospective study was to quantify the treatment dose during upper limb RT sessions using the data recorded by the rehabilitation robot. The measurement of a large amount of continuous activity data by a rehabilitation robot allowed the objective quantification of the internal dimension of the treatment dose administered; otherwise, this dimension is usually low in accessibility. The results showed that training, associating RT and occupational therapy, was characterized on the robot by a decrease in the time spent using the assist-as-needed modality and by an increase in both the mean number of repeated movements and the mean total distance traveled during the training time for all modalities pooled. In addition, the reduction in motor impairment, as measured by the FMA, was observed after 15 sessions of each therapy.

### 4.1. Measurement of Dimensions of Dose Using a Robotic Device?

This work shows the advantages of using technology to automatically record all data during exercise in robot-mediated therapy (input) and to measure changes in performance across sessions (output). The recent recommendations for the development of standardized studies in rehabilitation highlight the importance of providing a systematic, exhaustive description of the content of rehabilitation sessions, which also indicates the dose of the treatment administered [25]. This framework, which is validated by international experts, defines the multiple dimensions of the intervention dose. The variables measured by the robotic device in this study provide an instantaneous and simultaneous representation of the three characteristics of training, the internal dimensions: duration (mean actual practice time), difficulty (physical modality used), and intensity (mean number of repeated movements). The time spent actively training and the number of movements performed in each modality provide an objective description of one aspect of difficulty. In our opinion, the mean total distance traveled, which is not considered in the framework proposed, is an important variable that is specific to the pointing task performed during the RT. The distance between the starting point and the center of the target is one of the components of Fitts’ law. Altering target size and distance would provide another means of changing task difficulty [37,38,39]. However, the robotic system used in this study does not currently permit modulation of target size or distance. This could be considered by manufacturers to provide another means to modulate the difficulty of the exercise. To date, there is a random mode on the direction of the targets to be reached but always with the same distance and the same target size. Still in connection with the law of Fitts, it would be relevant to be able to influence the parameters of the distance to the target and the size of the target to make them vary randomly. This could further enhance motor learning by increasing contextual interference [40,41].

The description of the internal dimension of the intervention dose in this study is based on three parameters according to the training modality used: the mean actual practice time, the mean number of repeated movements, and the mean total distance traveled. The mean actual practice time is accurately recorded by the robotic system. However, the count of the number of movements is not entirely accurate. Only movements in which the target is reached are counted. Therefore, the mean number of repeated movements in the unassisted and against-resistance modalities may be underestimated if the patient does not reach the target in each attempt. This may explain the similar visual appearance of the curves for the mean number of repeated movements and the mean total distance traveled over time in Figure 3. Nevertheless, the patients performed on average more than 600 movements per session during RT. This number is comparable to that achieved by the patients who underwent the most intensive RT program in the study by Hsieh et al. (2012) [42].

### 4.2. A New Paradigm for a Combined and Progressive Approach to the Use of the Training Modalities?

Over the course of the rehabilitation program (with all modalities pooled), the mean number of repeated movements and the mean total distance traveled increased significantly, while the mean actual practice time remained constant. The mean actual practice time decreased only in the assist-as-needed modality. The patients thus covered an increasingly greater distance (number of movements multiplied by the distance to reach the target) while progressively using less robot assistance. The treatment intensity was thus increased by combining the quantitative (number of movements) and qualitative (motor difficulty of performing each of these movements) aspects of training. The paradigm applied, therefore, focused on the difficulty and intensity of movement performance, in contrast to traditional techniques, such as facilitation and neuromotor reprograming methods. A meta-analysis confirmed that repetitive task training has positive immediate and long-term (up to 6 months post-stroke) effects on upper and lower limb function [43].

The mean number of repeated movements and the mean total distance traveled changed over the course of the program, although not significantly: the mean number of repeated movements and the mean total distance traveled decreased in the assist-as-needed modality, while they increased progressively in the unassisted and against-resistance modalities. This demonstrates the importance of increasing the motor difficulty of the exercises by advancing the training modality. Although there is currently no consensus for the optimal use of training modalities to increase the difficulty of the exercises as the patient progresses [44], the use of the assist-as-needed modality alone is not recommended [31]: over-assisting movement during rehabilitation could have a deleterious effect on patient progress. Indeed, assistance could minimize the patient’s effort and reduce cortical activation [45]. It is important to administer unassisted exercises to ensure a sufficient level of motor difficulty [46]; the ultimate progression is the addition of resistance to the patient’s movement. Active voluntary movement is crucial for the recruitment of brain areas and to induce neuroplasticity by motor learning [47]. Most studies of robotic rehabilitation use only one control modality, often the assist-as-needed modality, except for the study by Stein et al. (2004), which combined the use of the assistance-as-needed modality with the counter-resistance modality [48]. To date, no studies have evaluated the impact of combining different modalities to increase the motor difficulty as the patient’s performance improves. It is beyond the scope of the present study to discuss the value of such an approach since the number of patients in each sub-group is small.

### 4.3. Optimizing Session Productivity?

The mean actual practice time of RT (exercises and games) per session in the present study was 34 ± 8 min of the 60 min session. There was, thus, an average loss of 43% of session time. Although the practice time (57%) was satisfactory compared to the percentage of active time reported in a literature review for conventional therapy sessions (between 2% and 10% in physiotherapy and between 23% and 70% in occupational therapy) [24], the percentage of lost time was far greater than that reported in a previous study that did not involve a robotic device (between 21% and 30% of an hour session, [49]). Even if the set-up time is not counted (−10 min), the proportion of lost time was high (32%).

RT should be provided in addition to conventional therapy: substituting conventional rehabilitation with RT does not improve outcomes more than conventional therapy alone [50]. The fact that a large proportion of session time is lost should, therefore, not call into question the concept of a combined program, but it is necessary to understand how to optimize rehabilitation time by using a robotic device. Providing additional daily rehabilitation time is one way to increase rehabilitation intensity. However, it is likely that 60 min of intensive rehabilitation with a robotic device is too long for patients. Although it has been shown in the literature that this type of intensive therapy is well tolerated [51,52,53], the mean actual practice time is rarely calculated.

In the present study, the lost time could be the rest time that is necessary to recover from the effort of each exercise. Future studies should attempt to characterize lost time as well as evaluate the influence of rest time during sessions on training with high motor difficulty. By shortening the session time, the lost time may be reduced without reducing the mean actual practice time. Adjusting the duration of RT sessions could, therefore, impact motor outcomes: controlling session duration as well as resting time would help to better control the dose delivered to patients.

### 4.4. What Are the Clinical Benefits of Combining RT with Conventional Upper Limb Therapy?

At the end of the combined upper limb rehabilitation program involving occupational therapy and RT, all sub-scores on the FMA had improved and the total FMA score (+11 points) exceeded the minimal clinically important difference [54]. Although a comparison with previous studies must be performed cautiously due to different demographics (discussed in the limitations section), the magnitude of recovery exceeded that expected for spontaneous recovery. The improvements in the FMA score are close to those reported after other intense rehabilitation programs involving the same robotic system [55,56]. The significant improvement in all the FMA sub-scores shows that the improvement was not limited to the joints that were principally trained by the robot. This is likely, at least partly, owing to the inclusion of conventional occupational therapy in the program. The repetitive and difficult training of shoulder and elbow joint movement by the robot was complemented by functional practice in conventional occupational therapy sessions, which included reaching and grasping tasks, as recommended by many studies, including the recent large RATULS study [57,58,59,60]. The results of the present study are, therefore, consistent with the literature and highlight the value of combining intense training of shoulder and elbow movements using a robotic device with functional training including the hand in occupational therapy. Such a “bottom-up” approach should enable improvements in motor function to be translated into increases in functional capacity [61].

### 4.5. Limitations

Methodologically, this work is a retrospective study, which could impact the outcomes observed. The demographic characteristics of our sample differ from those of studies that have reported motor recovery profiles following a stroke [2,4,62,63], and the mean age of the sample (n = 36; 59 ± 16 years) was lower than the mean age at stroke onset in France in 2014 [64]. The patients were also younger than those in the studies by Duncan et al. (2000) [5] (mean age 70 years) and Wade et al. (1983) [2] (mean age 67 years). This is important because the aging process has a negative impact on motor learning capacity [65]. Furthermore, the combined rehabilitation program was performed during the subacute phase of the stroke, a period during which the mechanisms of spontaneous plasticity are still actively at work. The improvement in impairment may therefore be, at least partly, the result of lesion-induced plasticity. Although the FMA is strongly related to a functional scale [66] (modified Frenchay Activities Index [67]), we did not evaluate function. It is essential that future studies include evaluations that cover all the domains of the International Classification of Function. This work presents a rare description of accurate data regarding the treatment actually administered to patients enrolled in the combined program. This in-depth exploration constitutes a first step in understanding the relationship between treatment dose and the effects on motor capacity.

## 5. Conclusions

This study highlights the unparalleled value of rehabilitation robots for describing as comprehensively as possible the dose of physical treatment administered to patients during rehabilitation sessions using a robotic device. This work tries to approach as well as possible the different dimensions of the dose delivered by a non-pharmacological treatment. It shows the significance of the standardized use of control modalities. To maintain a sufficient level of difficulty of the rehabilitation motor exercises as the patient progresses, it is possible to combine these modalities. Objectively, intensification is achieved through an increase in the mean number of repeated movements and the mean total distance traveled in all modalities and through a decrease in the mean actual practice time in the assist-as-needed modality. In coherence with the literature, this work shows again the significance of robotic devices used as adjuvant treatment to intensify and densify the management of patients in the subacute phase following a stroke.

## Figures and Tables

**Table 1:** Baseline characteristics of participants.

Participants (n)	36
Age (years)	59(16)
Female (n)	15
Side of paresis (n)	R (19), L (17)
Etiology (n)	I (25), H (11)
Time since the stroke at program initiation (days)	54 (26)
Duration of the program (days)	31 (11)
Initial FMA score (/66 pts)	23 (17)

Results are expressed as the mean (SD). R, right; L, left; I, ischemia; H, hemorrhage; FMA, Fugl–Meyer Assessment.

**Table 2:** Clinical outcome.

Fugl–Meyer	Pre-Intervention	Post-Intervention	Gain	*p*-Value
Overall (66 points)	23.1 (16.8)	34.3 (18.9)	11.2 (9.6)	<0.001
Shoulder/elbow (36 points)	14.6 (8.8)	20.9 (9.4)	6.3 (5.4)	<0.001
Wrist (10 points)	2.5 (3.4)	4.2 (3.8)	1.7 (2.1)	<0.001
Hand (14 points)	4.3 (4.5)	6.9 (5.2)	2.6 (3.3)	<0.001
Coordination velocity (6 points)	1.8 (1.6)	2.3 (1.8)	0.5 (1.2)	0.013

Results expressed as the mean (SD). In the first column, the total score and sub-scores are indicated with each corresponding maximal possible score in parentheses. Pre vs. Post: *p* < 0.05.

**Table 3:** Robot-based variables (all training modalities pooled) over 15 sessions of RT.

	Mean Actual Practice Time (%)	Mean Number of Repeated Movements	Mean Total Traveled Distance (cm)
Main effect *p*-value	ns	<0.001	<0.001
Session 1	47 (16)	624 (308)	6766 (4510)
Session 2	50 (14)	674 (264)	7294 (4437)
Session 3	49 (15)	676 (321)	7476 (4975)
Session 4	47 (14)	694 (282)	7422 (4436)
Session 5	46 (12)	725 (258)	7887 (4211)
Session 6	50 (13)	754 (318) ^a^	8508 (4775) ^a^
Session 7	50 (14)	772 (322) ^a^	9007 (5045) ^a^
Session 8	49 (13)	771 (336) ^a^	8991 (5131) ^a^
Session 9	51 (16)	797 (360) ^a,b,c^	9338 (5555) ^a,b,c,d^
Session 10	51 (15)	777 (362) ^a^	9403 (5494) ^a,b,c,d^
Session 11	51 (15)	805 (389) ^a^	9955 (5818) ^a,b,c,d,e^
Session 12	50 (14)	754 (342)	9096 (4941) ^a^
Session 13	52 (12)	806 (391) ^a^	10104 (5677) ^a,b,c,d,e^
Session 14	50 (13)	796 (364) ^a,b,c^	9999 (5123) ^a,b,c,d,e^
Session 15	46 (13)	764 (373) ^a^	9593 (5141) ^a,b,c,d^
Session 1–Session 15 changes	−1 (20)	140 (381)	2828 (4943)

Results are expressed as the mean (SD). ^a^ vs. Session 1: *p* < 0.05; ^b^ vs. Session 2: *p* < 0.05; ^c^ vs. Session 3: *p* < 0.05; ^d^ vs. Session 4: *p* < 0.05; ^e^ vs. Session 5: *p* < 0.05.

## Data Availability

All data are available in electronic format at the Centre de Réadaptation Fonctionnelle (CRF) Les Trois Soleils.

## Abbreviations

FMA	Fugl–Meyer Assessment
RT	Robot-assisted therapy
ANOVA	Analysis of variance
